# Striking Similarities in Fly and Vertebrate Olfactory Network Formation

**DOI:** 10.1371/journal.pbio.1001401

**Published:** 2012-10-02

**Authors:** Janelle Weaver

**Affiliations:** Freelance Science Writer, Glenwood Springs, Colorado, United States of America

**Figure pbio-1001401-g001:**
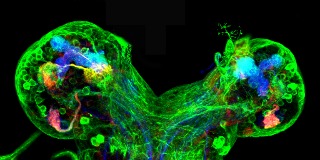
Olfactory projection neurons (left, red) are absent following early unilateral ablation of presynaptic sensory neurons (right).

[Fig pbio-1001401-g001]Olfaction is a primitive sensory ability that is common to vertebrates and insects, but the neural circuits that underlie the ability to smell are very complex. Before an odor is recognized, it has to be processed through several stages by neurons in the olfactory system. Olfactory sensory neurons (OSNs), which detect odors at the first stage of processing, form precise connections with cells in brain structures called glomeruli, whose specific activation patterns contain information about odor identity. Despite the common design principles in vertebrates and insects, there are important differences. OSNs drive the formation of glomeruli in vertebrates, but cells in glomeruli develop independently of OSNs in adult insects.

This week in *PLOS Biology*, Lucia Prieto-Godino of the University of Cambridge and her collaborators report the results of the first systematic investigation of how the olfactory network assembles in fruit fly embryos. They found that the activity of OSNs is crucial for the proper formation of connections in the olfactory system during these early stages of development. The findings reveal surprising similarities between vertebrates and insect embryos in the formation of olfactory networks.

Specifically, they found that projection neurons in the glomeruli of fruit fly embryos only formed extensions that would eventually connect with other neurons after being contacted by OSNs, unlike the pattern seen in adult fruit flies. Projection neurons were more likely to survive when they were contacted by OSNs, and the activity of OSNs controlled the growth of extensions formed by projection neurons.

OSN activity also controlled how OSNs formed connections with cells in glomeruli. When the researchers genetically silenced OSN activity, they found that the OSNs formed connections that extended across a broader-than-normal swath of glomeruli, similar to previous findings in vertebrates. This suggests that OSN activity is crucial for the normal wiring of the olfactory network.

The study reveals new insights into the development of the olfactory network in insect embryos and shows that OSN activity plays a key role in guiding the development of the olfactory circuitry. Surprisingly, these patterns resemble those observed in vertebrates, but not adult fruit flies. Because neural circuits in other sensory and motor systems share similar properties, the findings may represent general mechanisms that underlie the development of networks in the nervous system.

**Prieto-Godino LL, Diegelmann S, Bate M (2012) Embryonic Origin of Olfactory Circuitry in** ***Drosophila*** **: Contact and Activity-Mediated Interactions Pattern Connectivity in the Antennal Lobe. doi:10.1371/journal.pbio.1001400**

